# Antimicrobial peptides: An annotated selection of World Wide Web sites relevant to the topics in microbial biotechnology

**DOI:** 10.1111/1751-7915.13368

**Published:** 2018-12-21

**Authors:** Lawrence P. Wackett

**Affiliations:** ^1^ Department of Biochemistry, Molecular Biology and Biophysics BioTechnology Institute University of Minnesota St. Paul MN 55108 USA

## Compendium of antimicrobial peptide databases

https://omictools.com/antimicrobial-peptide-data-category

This Omic tool listing contains an annotated set of antimicrobial databases. Antimicrobial peptides are increasingly deemed to be important in biotechnology for their animal and human therapeutic potential.

## The anti‐microbial peptide database

http://aps.unmc.edu/AP/structure.php

This site contains a database of peptides, prediction tools and classification schema. Overall, it is a comprehensive resource on the topic.

## Collection of anti‐microbial peptides

http://www.camp.bicnirrh.res.in

This collection focuses on sequence patterns and HMMs for antimicrobial peptides and provides links to major databases such as UniProt and PubMed.

## Peptaibol database

http://www.cryst.bbk.ac.uk/peptaibol

This site focuses on a specific class of antimicrobial peptides known as peptaibols. These peptides are typically derived from fungi.

## Database AMP

http://csb.cse.yzu.edu.tw/dbAMP/

This database is particularly useful for identifying antimicrobial peptides using transcriptomic and proteomic data.

## Database of antimicrobial activity

https://dbaasp.org

This database specializes in modeling structures of antimicrobial peptides to help predict therapeutic potential for these compounds.

## Tachystatin A structure

http://www.rcsb.org/structure/1CIX

This record in the Protein DataBank has information and structural coordinates for techystatin A, an antimicrobial peptide from horseshoe crab.

## Four structural classes of antimicrobial peptides

https://www.researchgate.net/figure/Four-structural-classes-of-antimicrobial-peptides-A-a-helical-structure-of-human_fig1_281555477

This page from the Research Gate site posts information on a specific collection of antimicrobial peptides from animal origin.

## Structure of anoplin in micelles

https://www.rcsb.org/structure/2MJQ

This record in the Protein DataBank has information and structural coordinates for antimicrobial peptide anoplin that is embedded in diphosphatidylcholine micelles. Anoplin is an antimicrobial peptide from a species of wasp.

## Antimicrobial peptides review

https://www.mdpi.com/2218-273X/8/1/4/htm

This review article explores antimicrobial peptides, especially from the standpoint of providing a replacement of some antibiotic therapies where resistance is rising.

## Antimicrobial peptides for wounds

https://www.frontiersin.org/articles/10.3389/fphar.2018.00281/full

This article discusses the use of antimicrobial peptides for surface injuries such as skin wounds.

## General Probiotics

http://gprobiotics.com

General Protobiotics is a biotechnology company exploring commercial use of antimicrobial peptides in the poultry industry, as a replacement for antibiotics. The use of antibiotics in animal agriculture is increasingly being constrained.

## Selling antimicrobial peptides: Hycult Biotech

https://www.hycultbiotech.com/products/innate/antimicrobialpeptides

This biotechnology company sells a variety of antimicrobial peptides, largely for research purposes.

## Antimicrobial peptides: Gordon Research Conference

https://www.grc.org/antimicrobial-peptides-conference/2019/

This Gordon Research Conference is designed to host high‐level discussion of antimicrobial peptides, with a particular focus on their potential efficacy in human and veterinary medicine.

## Designing antibacterial peptides

https://www.ncbi.nlm.nih.gov/pmc/articles/PMC5829097/

An important area of antimicrobial peptide research is understanding structure/function correlations. This is discussed here in the context of designing more effective, faster‐acting peptides.

## Molecular simulations of antimicrobial peptides

https://www.ncbi.nlm.nih.gov/pmc/articles/PMC2989729/

Antimicrobial peptides largely exert their effects in membranes. This study uses molecular dynamics simulations to better understand the interactions between the peptides and specific membrane components.

## Antimicrobial peptides: Wikipedia

https://en.wikipedia.org/wiki/Antimicrobial_peptides

This Wikipedia entry provides a particularly good tutorial on many aspects of antimicrobial peptides.

## Structure/function of antimicrobial peptides

https://www.sciencedirect.com/science/article/pii/S1876162318300282

This review focuses on biophysical and computational methods for studying antimicrobial peptides.

